# Sustainable rare diseases business and drug access: no time for misconceptions

**DOI:** 10.1186/1750-1172-8-109

**Published:** 2013-07-23

**Authors:** Pierrick Rollet, Adrien Lemoine, Marc Dunoyer

**Affiliations:** 1 GSK Rare Diseases, 980 great west road, Brentford, Middlesex TW8 9GS, UK

## Abstract

Legislative incentives enacted in Europe through the Regulation (EC) No. 141/2000 to incentivize orphan drug development have over the last 12 years constituted a powerful impetus toward R&D directed at the rare diseases population.

However, despite therapeutic promises contained in these projects and significant economic impact linked to burgeoning R&D expenditures, the affordability and value of OMPs has become a topic of health policy debate in Europe fueled by the perception that OMPs have high acquisition costs, and by misconceptions around pricing dynamics and rare-diseases business models. In order to maintain sustainable patient access to new and innovative therapies, it is essential to address these misconceptions, and to ensure the successful continuation of a dynamic OMPs R&D within rare-diseases public health policy.

Misconceptions abound regarding the pricing of rare diseases drugs and reflect a poor appreciation of the R&D model and the affordability and value of OMPs.

Simulation of potential financial returns of small medium sized rare diseases companies focusing on high priced drugs show that their economic returns are likely to be close to their cost of capital. Research in rare diseases is a challenging endeavour characterised by high fixed costs in which companies accrue substantial costs for several years before potentially generating returns from the fruits of their investments. Although heavily dependent upon R&D capabilities of each individual company or R&D organization, continuous flow of R&D financial investment should allow industry to increasingly include efficiencies in research and development in cost considerations to its customers. Industry should also pro-actively work on facilitating development of a specific value based pricing approach to help understanding what constitute value in rare diseases. Policy makers must reward innovation based upon unmet need and patient outcome. Broader understanding by clinicians, the public, and policy makers of the complexity of clinical programs to deliver OMPs to market is required to better comprehend the decisions needed and made by industry. In parallel, an overt effort to consider the impact of public policies on R&D investments is key to enable policy makers to better reconcile the incentives provided by public policy decisions and companies investments decisions in a more positive manner.

## Introduction

Orphan Medicinal Products (OMPs) are intended for the diagnosis, prevention or treatment of serious, rare diseases that substantially affect life expectancy, physical and social functioning of patients and their families [1-4]. Since the introduction of EU OMP regulation in the year 2000, there has been a powerful impetus for research and development (R&D) in the rare diseases field and there are now more than 1,000 medicines with OMP designation. As a result, OMP R&D expenditures in the EU have more than tripled between 2000 and 2008 and overall employment in all departments of companies working on OMPs have more than doubled between 2000 and 2008 driven by increasing number of R&D activities and staff located in the EU [5]. The dramatic increase in OMP designations shows a desire to advance scientific understanding of disease mechanisms and a willingness to seek new drugs that offer therapeutic benefit to patients and families for whom no effective treatment options are available.

However, despite the promises offered by this surge in orphan designations and corresponding R&D expenditures, the affordability and value of OMPs has become a topic of health policy debate in Europe [6], fueled by the perception that OMPs have high acquisition costs and by questions-misconceptions around pricing dynamics and rare diseases business models. These commonly encountered misconceptions are:

1. OMPs are high priced and are significantly more expensive than non orphan drugs

2. Budget impact of OMPs is high and high priced OMPs exacerbates affordability problem for health care budgets

3. Factors affecting R&D investments and returns are more favorable for OMPs

4. Cost of Manufacturing should determine the fair price of OMPs

5. Rare diseases companies are making excessive financial returns thanks to high priced drugs

In order to maintain sustainable patient access to new and innovative therapies, it is essential to address these misconceptions, and to ensure the successful continuation of dynamic OMPs R&D within rare-diseases public health policy. Failure to do so will result in an “innovation pile-up”, such that patients with rare diseases in Europe will not be able to access the benefits afforded by scientific innovation. Previous authors have contributed to address some of the common questions related to orphan drug development and its regulation [7]. This article aims at providing a complementary industry perspective on some of the key questions and misconceptions related to value-pricing and the business model for rare diseases therapies, with the aim of facilitating discussion and future progress in the field.

## OMPs are high priced and are significantly more expensive than non-orphan drugs?

A common perception is that many rare-disease drugs are high priced and offer higher prices than non orphan drugs [8]. Using public list prices [9] and approved EMA dosing schedule [10], a thorough comparative analysis of annual weighted treatment costs of all OMPs approved in Europe as of December 2012, shows a more complex picture.

First, a review of the profile of all 89 OMPs approved in EU between 2000 and 2012 reveals that rare diseases treatments offer is not uniform but can be regrouped along 4 homogeneous categories.

**Figure 1:**
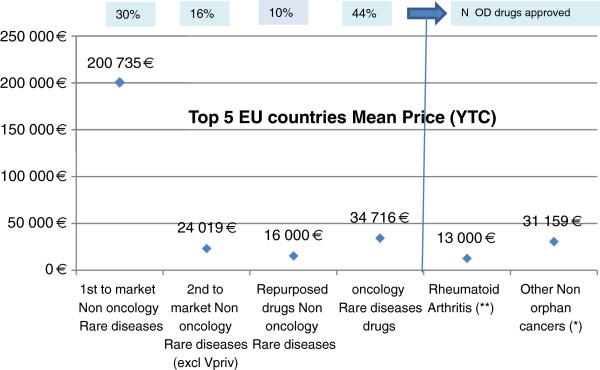
**A comparison of the top 5 European countries’ mean price treatments for OMPs and mean price treatments for a select sample of non-rare disease hospital-specialty drugs *(%indicate the share percentage of each category in all approved OMPs in Europe as of December 2012*).** (*)Mabcampath, Mabthera ,Tasigna ,Velcade,Sutent, Herceptin, Avastin, Iressa / (**) Humira, Enbrel, Remicade, Mabthera.

Orphan oncology drugs do represent a first category and the majority of approved OMPs in Europe (44%-Figure 1 below). These treatments often indicated across multiple cancer indications present, in their majority, efficacy data across consistent clinical end points such as overall survival or progression free survival. Such homogeneous data set facilitates comparative value definition and pricing comparison across oncology treatments.

A second category includes repurposed drugs which, by definition, originate from an extended development in a rare disease indication of an established product approved for a common disease indication. They only represent 10% of approved OMPs in EU and from a pricing perspective, the initial common disease indication is often used as benchmark. A third category is the 2^nd^ to market rare diseases treatments, representing 16% of the approved OMPs in EU and which share with the latter group the feature of available price comparator against which to position value and price of a new treatment.

A last category covers first to market, non oncology OMPs. These drugs represent 30% of the approved OMPs in EU and are characterized by no approved treatments as standard of care at time of approval. In their majority, they target chronic, degenerative, very rare diseases (average population size of 5 000 patients across main five EU countries). These drugs are also representative of heterogonous clinical circumstances. Data packages are diverse with end points supporting efficacy of these treatments neither consistent nor comparable across drugs. As a result, they are the category of rare diseases treatments where pricing and reimbursement decisions are often the hardest to define and justify.

We conducted a comparison of the mean price of the above described OMPs categories versus annual treatment cost of a sample of non-rare diseases hospital specialty drugs across the top 5 European countries (France, Germany, Italy, Spain, UK). List of drugs is described in detail in the online Technical Additional file 1.

Our comparative pricing analysis shows that price points (yearly patient costs per course) for first-to-market non-oncology rare disease treatments are in the high range with the average price per patient per year of 200,000 Euros. In contrast, non-oncology repurposed and second-to-market OMPs are characterized by price points in the range of 16,000 – 24,000 Euros per patient which compares to mean yearly treatment costs of 13.000 Euros for Rheumatoid Arthritis non orphan hospital drugs approved in EU. The average cost per cycle for oncology OMPs is in the range of 35,000 Euros per patient and similar to non-orphan oncology drugs (Figure 1 below).

The methodologies used for above analysis deserve a few comments. First, the geographic scope of our analysis is most populated EU 5 countries which present homogeneous economic purchasing power [11], hence a relevant base for pricing comparison and analysis.

Second, the presented average price of EU approved second to market OMPs exclude one drug “velaglucerase-alfa”. The rationale is that although “velaglucerase-alfa” is on average priced at a 4% discount (list price) across EU 5 countries versus the reference treatment “Imiglucerase” for a patient of similar weight in the same disease indication, “velaglucerase-alfa” represent, from price point perspective, a clear outlier against other approved 2^nd^ to market OMPs. If including “velaglucerase-alfa” in the analysis, the average price of 2^nd^ to market OMPs approved across most populated EU 5 countries would increase to 56–000 Euros per patient per year.

Third, rheumatoid arthritis treatments are a very relevant non rare diseases category against which to benchmark prices of non oncology rare diseases drugs because of their very similar profile and delivery setting in comparison to OMPs. They are hospital drugs prescribed and delivered in a similar high specialized hospital setting. Similar to majority of rare diseases drugs, they also target chronic degenerative diseases and often “compete” at hospital level with budget of rare diseases drugs [12]. In addition, approved across multiple indications (in majority non orphan) but indicated in USA with orphan status in only a few indications such as paediatric ideopathic RA (Adalimumab), some authors are using above $1 billion sales level generated by these RA drugs across all indications to present and position them as “orphan sales blockbusters” and main OMPs driving potential affordability challenges [13,14]. For all these reasons, a pricing comparison between these drugs and rare diseases treatments is therefore relevant. For non-orphan oncology drugs, the selected comparison base is oncology treatments which, across their multiple indications, represent the majority part (2/3) of oncology volume of prescriptions in Europe [10].

In conclusion, against all common perceptions, OMP prices are clearly differentiated along rare-diseases product-categories. The majority of approved Orphan drugs (70%) have lower prices or in the range of other non-rare diseases specialty hospital drugs. Only 30% of all approved OMPS in EU have much higher prices.

## Budget impact of OMPs is high and high priced OMPs exacerbates affordability problem for health care budgets?

A commonly encountered comment is that rare diseases drugs have high budget impact and that that OMPs with high prices described in previous chapter would constitute a substantial affordability challenge to healthcare budgets.

Current context of economic crisis in Europe clearly obligates and justifies the need to pay close attention to drugs budget impact since a substantial part of these costs are financed by national healthcare insurance systems. For rare diseases drugs, their budget impact in Europe is in fact low, in the range of 1% -4.6% of total drug spending [15-17]. OMPs with the highest price levels are also those that turn out to have the smallest budget impact. This is confirmed by an analysis from France, the country in Europe with the largest number of reimbursed OMPs, where orphan oncology drugs were accounting for more than 60% of total orphan drug spending in 2010 and they were also the drugs with the lowest price points among all OMPs [16]. The population size to be treated rather than individual drug price influences the budget impact of rare diseases treatments and drives affordability.

Importantly, the budget impact of OMPs is concentrated in a very small number of drugs. France again serves as a good case study. A recent AFM (*) report showed that five drugs accounted for about 50% of the French OMP budget impact in 2010 [16]. In terms of future dynamics, recent research indicates that the budget impact of OMPs in the EU is likely to plateau in next 5 years, to a level of 4%-6% of total drug spending, with this plateau effect driven by patent-losses among the very few drugs that contribute the most to the budget impact [15].

Increasing economic constraints in Europe make policy makers and healthcare managers concerned that budget impact of OMPs is prohibitive and that OMPs with high prices could constitute a substantial affordability challenge to healthcare budgets [18]. Such concerns are legitimate and it is key that the rationale and appropriate use of OMPs balance the interests of all stakeholders amid the growing cost pressures and uncertainties that permeate implementation of a given drug post launch.

Against these concerns, publicly available national statistics show however that OMP budget impact is low due to small population size and will likely plateau in next 5 years. Budget impact is also concentrated in a small number of drugs, mostly oncology treatments with prices no different to non orphan cancer products. OMPs with the highest price levels are in addition those that turn out to have the smallest budget impact.

The concerns that OMPs with high prices and their budget impact poses a substantial affordability budget challenge to healthcare systems do not therefore appear to be justified.

In this evolving environment, new models for handling uncertainty and ensuring value continue to be developed with a growing interest among payers for agreements that involve country-specific innovative performance-based or “risk-sharing” elements [19,20]. This means that to overcome potential tension between funding of OMPs and affordability, concentration of budget impact in a few rare diseases drugs makes potentially any significant affordability issue more easily “localized” while providing potential incentive to develop mechanisms that can help control costs without negatively impacting their patient populations.

(*) Association Française contre les Myopathies.

## Factors affecting R&D investments and returns are more favorable for OMPs?

Following US Orphan Drug Act (ODA) introduced in 1983, a significant number of countries in Europe and Australasia have established OMP regulatory pathways. These specific legislative incentives have created favorable environment to invest in R&D programs for OMPs. This has materialized for example in a significant increase in number of OMP designation approved in EU & USA [21]. Arguing of these specific legislative incentives, some authors have commented that factors affecting R&D investments and returns are more favorable for OMPs [22,23].

In reality, these perceptions overlook a number of structural factors at play that affect pharmaceutical R&D investments across diseases, including in rare diseases. A dynamic analysis of main constituents that influence R&D programs and economic returns for a new medicine offers a more contrasted perspective.

**Figure 2:**
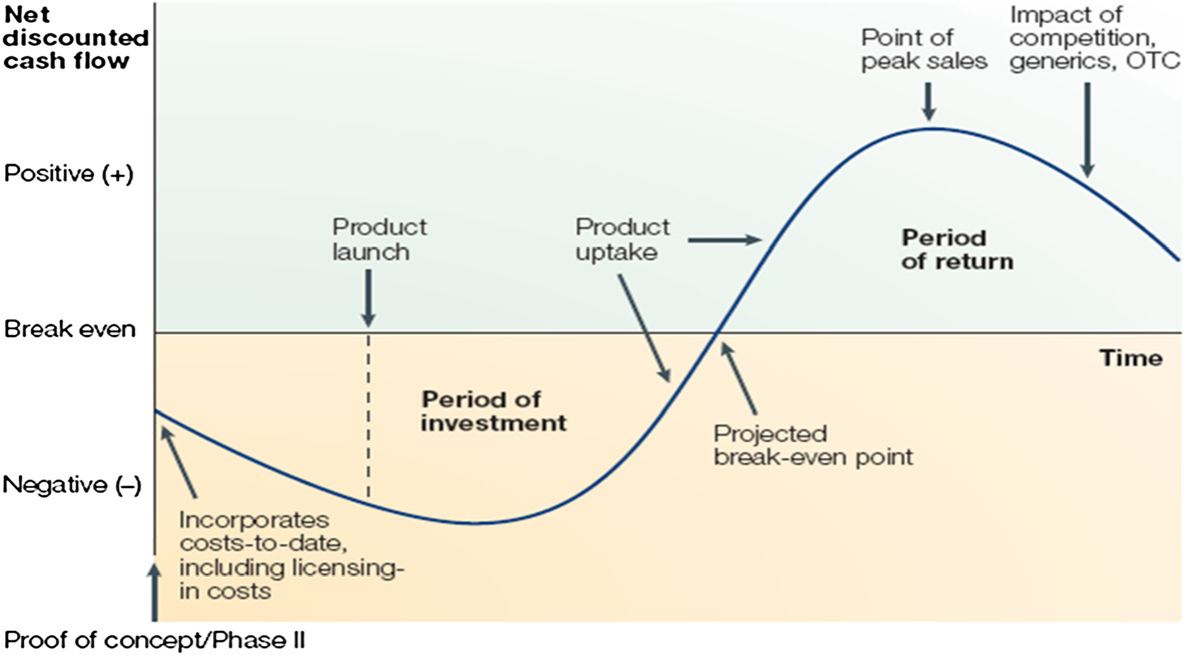
Schematic view of investment and return periods for the development of pharmaceuticals.

First, the development cycle of any drug, including OMPs, obeys the same structural features of a lengthy process in which industry accrues substantial costs for several years before potentially generating revenues and returns from the fruits of R&D (See Figure 2 below). Time-value of money, together with other costs impacting generation of net cash flows over the life cycle of a drug is therefore a key constituent of R&D returns of any drugs, including OMPs.

Second, four drivers - success rates, development times, R&D out-of-pocket costs and cost of capital overall impact capitalized R&D costs and influence economic returns for developing any new medicine [24,25]. An analysis from published information on how these four drivers behave in the specific context of rare diseases R&D models shows that that no generic conclusion can be drawn on potential more favorable R&D conditions for OMPs.

### Success rates

Attrition rate, defined as the proportion of failures out of the total number of projects entering any given stage of R&D, is the most important driver of R&D cost [24,25]. Recent research shows that reducing Phase II and Phase III failure rates by 25% and 20% respectively would have the greatest effect on decreasing the cost of developing a new molecule, reducing costs by around 37% [25].

Rare diseases share specific features such as well identified pathways for monogenic diseases or clinical development for repurposed drugs already tested in other indications that are likely to favor higher probability of success.

In practice however, some authors have recently documented a lower approval rate of Marketing Approval Authorizations (MAA) for Orphan medicinal products (59,8%) versus non-OMPs (75.6%) during the 1995–2007 period [26]. This might be explained for a large majority of OMP programs and those in particular targeting diseases with no available treatments, by the scarcity of available patient pool, the heterogeneous populations and the difficulty in identifying validated clinical end points that are structural hurdles contributing to increased risk of failures. This is consistent in addition with the features of a majority of OMP clinical programs, which, by studying treatments in patients with chronic-degenerative, potentially fatal diseases and by favouring investigation of drugs with novel mechanisms of action, are known to predict an increased risk of failure [24].

Some research on Pharmaceutical R&D productivity have revealed that success rate remains a function of each single company or R&D organization fostering its scientific creativity and leveraging a differentiated portfolio strategy and R&D capabilities [24,25]. The same variability and dynamics across companies are likely to apply to rare diseases. Recent publications have however evidenced emergence of some potential differentiated specific success drivers. First, protocol assistance provided by EU regulation 141/2000 provides a valuable guidance to companies developing OMPs and obtaining-complying with such scientific advice has been recently documented as a critical factor for a successful Marketing Approval Authorization (MAA) [26]. Second, company size, while a significant predictor of compliance with scientific advice was also found to be an important independent predictor of outcome of a MAA [26]. This is consistent with findings of Danzon et al. which in a previous publication had shown that a firm’s development experience, measured by the number of compounds with which the firm was involved as an originator, had considerable positive effect in phase 2-phase 3 development success showing that experience matters for the larger and more complex phase 2 and phase 3 trials [27]. In summary, OMP have historically a lower MAA success rate than non orphan treatments [26]. Success rate variability across therapeutic areas and across companies are likely to apply to rare diseases but documented productivity enhancing effects of alliances and scientific advice regulatory incentives suggest that potential differentiated factors exist for higher success rates of Marketing Approval Authorization (MAA) in this therapeutic space.

### Development times

The average time from discovery to drug registration is 12–13 years [25]. While the number of patients typically included in clinical trials for rare diseases is smaller than in more common diseases [22], recent research has shown that the overall clinical development time (Phase I to Phase III) is similar for orphan and non-orphan programmes at about 5.7 years [28]. One possible explanation for this may be the complexity of therapeutic R&D in rare diseases. Small patient populations present practical difficulties for trial recruitment. In addition, as illustrated by contrasted outcomes across EU and USA of recent regulatory file submission of the drugs pirfenidone, tafamidis or mipomersen [21], regulatory requirements across agencies remain yet to be harmonized. Combined together, these factors can lengthen development times. In addition, trade-offs may be occurring between time and success rate—for example, longer Phase II development times that allow for additional data generation before deciding whether to advance to the next phase might produce higher success rates in Phase III but lengthen the R&D process overall and may slow patient access to therapies in areas where no treatment is available. In summary, development times are on average broadly similar for orphan and non-orphan and do not appear to be more favorable for OMPs.

### Out-of-pocket costs

Some authors have conjectured that clinical development in rare diseases should be less costly because smaller patient populations make clinical trial out-of-pocket costs lower than those that would be needed to conduct trials in more common diseases, and they consider that this constitutes a major driver of greater profitability for OMPs [22,23]. In rare diseases, although number of patients in clinical trials is smaller [23], often the complexity of clinical trials is greater, due to factors such as a partial or incomplete understanding of the natural history of disease, or the large cost variability depending on the product origin (e.g. biologic compounds have higher out-of-pocket costs than non-biologics [24]). Travel costs linked to difficulty of finding patients, the often required high numbers of different clinical trial sites or the loss of valuable experience and expertise over the course of a trial that results from high turnover in CROs clinical teams and longer timelines are additional specific cost drivers in rare diseases which can make performing clinical trials more expensive on a per patient basis than would be the case in more common and better understood diseases.

**Figure 3:**
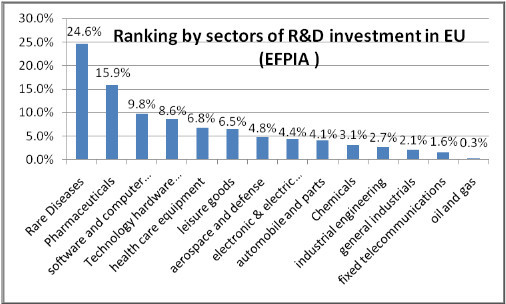
Ranking by sector of R&D investment in the EU (EFPIA **)). (**) European Federation of Pharmaceutical Industries and Associations.

Another consideration is to look at how rare diseases companies compare to other industries and general pharmaceutical industry in their invested out of pocket R&D cost relative to their revenues. The pharmaceutical industry is a competitive industry and reinvests about 16% of its revenues in R&D, giving it the highest R&D intensity in Europe when compared with other industries today Figure 3.

Benchmarking Rare diseases companies described in chapter 1 that focus on high priced first to market non oncology drugs, these companies reinvest an even higher share of their revenues in R&D , reinvesting 25% of their revenues, or 1.5 times more, than the average for the pharmaceutical sector [29]. The business model of these companies is associated with high priced drugs to counter balance the large focus on innovation and very rare populations targeted. For Rare diseases companies with such a business focus on high priced rare diseases drugs, the high invested out of pocket R&D costs relative to generated revenues reflect a riskier business and strategy model.

### Cost of capital

Capitalized cost is the standard accounting treatment for long-term investments. Given the timescales and challenges in developing a new medicine, the total cost of a successful drug needs to account for both failures and for the cost of capital. The cost of capital of a company measures the average cost of financing the company (via debt or equity), and represents at an investment project level the minimum return that needs to be generated to break even.

The long timescales of pharmaceutical R&D mean that the estimated R&D cost of a new drug is highly sensitive to the cost of capital applied because R&D costs, on average, are incurred (including for failed projects) many years before any revenue is earned to recover them. Recent studies show that approximately 33% to 50% of the total R&D cost of a new medicine is due to the cost of capitalizing R&D expenditures [24,25].

Through the cost of capital, financial investors require a risk premium for holding equity or lending money to a particular company and its R&D portfolio. As a result, cost of capital for small medium biotech enterprises is often quoted higher than for larger pharmaceutical companies and in the range of 11% to 14% in real terms (inflation adjusted) [30]. The 11%-14% cost of capital range is a reasonable estimate for the rare diseases industry, where the structure is heavily composed of small and medium size enterprises.

In summary, the financial burden of R&D expenditures for the SMEs developing OMPs is higher on a euro-for-euro basis than for the established large pharmaceutical companies. Cost of capital will also vary company by company reflecting relative riskiness of business model and portfolio structure.

Overall, an analysis from most recent published information shows that factors affecting R&D investments are not proven to be more favorable for OMPs. They remain heavily dependent upon how, each individual company or given R&D organization working in rare diseases could be better in fostering its scientific creativity or leveraging a differentiated portfolio strategy and R&D capabilities that could enhance pipeline productivity and success.

## Cost of manufacturing should determine the fair price of OMPs?

Arguing of specific OMP legislative incentives available in EU, a commonly encountered perception is that cost of manufacturing should be the base to determine a fair price of OMPs.

In real world, a balanced perspective on price setting components of a new medicine, including rare diseases drugs, requires a clear understanding of the dynamics associated with R&D investments in pharmaceuticals and on that respect, price setting constituents are no different for rare diseases drugs than those of other more common diseases.

To remain viable for the long term, pharmaceutical companies must make a profit in order to be able to continue to reinvest in the development of new medicines for complex conditions. Today’s profits pay for research and development of tomorrow’s medicines. A major factor driving profit and price setting of any drug, including OMP, is the unique R&D and financial investment process required to bring a product to market (see chapter 2). Such a process comprises the constituents that affect the financial viability of any individual R&D investments decision in the 12–13 years timeframe required to bring a drug from discovery to successful registration (see chapter 2). It can therefore result into differentiated drug prices. For example, the comparatively low prices of repurposed rare diseases drugs (see chapter 1) probably reflect less demanding clinical development requirements for this drug category as safety profile of these drugs is often already partly studied in other indications.

Although academic exchanges are ongoing on which costs of R&D to integrate into investment decisions [31], economically, a balanced drug price for any company investing in time consuming risky pharmaceutical development cycle, including for rare diseases, is one in which the proposed selling price of an individual drug allows shareholders to gain a return on their portfolio investment that is comparable to investment returns that are available to them in other industries or enterprises.

Price setting also includes other factors such as the value of the treatment and its budget-impact economic-justification to payers. Furthermore, prices are set in negotiations with purchasing parties in the context of regulated- government established processes and a final price is influenced by the result of the negotiations with governments, insurers and wholesalers.

In conclusion, price setting of any pharmaceutical product is not simply a function of its cost of manufacturing but multi-factorial across notably the time-value of money and other costs impacting generation of net cash flows over the life cycle of a drug.

## Rare diseases companies are making excessive financial returns thanks to high priced drugs?

Some authors have chosen to highlight what they see as the excessive financial returns generated by high priced OMPs [13], which often belong to earlier described first to market, non oncology category. Against this perspective, it should be pointed out that if a pharmaceutical or biotech company engaged in rare-diseases R&D is to recoup its R&D costs and generate profit, this investment will also inevitably need to be recovered through lower sales volume, given the structural limited population size for the product.

A simple approach to simulate current R&D return is to determine the required sales compounded annual growth rates to achieve a pre-determined return matching at least the cost of capital threshold, to enable break even (ie, making neither profits nor losses). We therefore analyzed 10 emblematic small medium size rare diseases enterprises which business model is focused on high priced first to market non oncology products described in chapter 1.

To remain viable for the long term, any R&D spend that pharmaceutical companies make on any given year must be recouped (in profits resulting from resulting sales) at a chosen fixed point in the future. Using a 10 year time lag, assuming a 11% rate of return (risk adjusted cost of capital) as per estimates of recent publications [24], it follows that every 100 M $ of R&D investment (corresponding to a net financial charge of 65 M $ for profitable companies, assuming a 35% profit tax rate) must produce incremental post tax profit of 185 M $ (ie 65 X 1.11^10) 10 years later. This is equivalent to around 284 M $ pretax profit. If a trading margin on year 10 sales of 40% is assumed (excluding R&D expenditure), it follows that 100 M $ of R&D expenditure must produce incremental sales of 473 M $ ten years later. With a cost of capital at 14%, it transpires that 100 M $ of R&D spend must generate 10 years later sales of 618 M $. Detailed calculations are described in the online table Additional file 2.

The aggregate R&D expenditure for the universe of 10 selected rare diseases companies was 2.9 Bio $ in 2012 [29]. To generate a return of 11% to 14% on this investment requires respective additional sales of 21 Bio $ and 27 Bio $ in the year 2021. These companies had cumulated sales of rare diseases products of 13.6 Bio $ in 2012. Our life-cycle model suggests that to achieve 11% to 14% return, this corresponds to 5% and 8% compound sales growth per annum for the 10 companies over 10 years. This compares with current 11.8% per annum compounded sales growth achieved over the last 5 years for these 10 companies or, considering structural plateau effect that follows early years of any new product launch, a 10 year sales growth pattern close to a 5%-10% range.

A simple simulation using the 10-year pay-back method shows therefore that the aggregate R&D returns from rare diseases companies are likely to be close to estimated cost of capital of these companies. This is consistent with comments made by Shire CEO in a recent interview referring to returns in the magnitude of 2-3% above the cost of capital of the company [32].

Policy approaches to incentivize innovation are complex. A valuable incentive exists for industry to invest in the development and marketing of orphan medicinal products through the 10 years market exclusivity provided by regulation (EC) No 141/2000 on orphan medicinal products [33]. The intended effect of this regulation is to extend the effective patent protection and to raise expected monetary reward to rare diseases drug research and thus encourage entry. Against some perceptions, market exclusivity granted in regulation (EC) No 141/2000 to an orphan medicinal product does not prevent the marketing of other Orphan medicinal product which, although similar to the orphan medicinal product already authorised, would be safer, more effective or otherwise clinically superior [31].

Current context of economic constraints in Europe however justifies the need to pay close attention to the rationale of maintaining such incentive in the context of potential return on investments of companies offering high priced drugs. Simulation of potential financial returns of small medium sized companies focusing on high priced rare diseases treatments shows that their returns are likely to be close to their cost of capital. When linking financial returns to drug prices, it also infers that the price charged for rare diseases treatments targeting very rare disorders must be seen in context of the rarity of the condition, the number of patients treated, and the R&D financial investment incurred. Whether the prices are justified in regard to the medical/social value of the OMPs is a different but critical matter not debated in this analysis.

## Outlook and conclusion

Misconceptions abound regarding the pricing of rare diseases drugs and reflect a poor appreciation of the R&D model and the affordability and value of OMPs. It is critical that multiple stakeholders understand and address these misconceptions in order to safeguard the overarching values of universality, equity, solidarity that are necessary to maintain equitable access to treatments for rare diseases.

Rare diseases remain unchartered territory and pose challenges for drug developers and to policy makers alike. Drug development in this arena focuses in the majority of programs on exploring new treatment targets using innovative technologies. Small size population makes research in rare diseases a challenging endeavor.

To ensure the successful continuation of dynamic OMPs R&D within rare-diseases public health policy, policy makers must reward innovation based upon unmet need and patient outcome. Policies that inflate research costs or protract the time from discovery to market increase the size of investments required for R&D.

At company level, technology platforms, diseases franchises, global reach are potential levers to sustain profitable development of OMPs. Simulation of potential financial returns of small medium sized rare diseases companies focusing on high priced drugs show that their economic returns are likely to be close to their cost of capital. Although heavily dependent upon R&D capabilities of each individual company or R&D organization, continuous flow of R&D financial investment should allow industry to increasingly include efficiencies in research and development in cost considerations to its customers.

Industry should also encourage development of a specific OMP value based approach embedded into a specific policy framework. Although an important component, it is not sustainable for the industry to continue only justifying value and prices of rare diseases treatments on the sole basis of the rarity of a condition. A consistent value definition that could mirror the significant benefit criterion would offer a possible construct for more comprehensive guidance to pricing and reimbursement decision-making, while helping to send appropriate signals as to priorities for future investment in R&D.

A broader understanding by clinicians, the public, and policy makers of the complexity of clinical programs required to deliver OMPs to market is required for stakeholders to better comprehend the decisions required and made by industry. There is no silver bullet. The answer to achieving improved sustainable patient access to new and innovative therapies requires solid rare-diseases public health policy founded on early dialogue, understanding and education.

## Competing interests

All co-authors were GSK employees at the time of the manuscript.

## Authors’ contributions

PR conceived the article, carried out the literature search, organized the sequence alignment and drafted the manuscript. AL participated in the design of the article and in the sequence alignment, provided comments in the manuscript, complemented the literature search. MD participated in the design of the article and coordination, helped to draft the various versions of the manuscript. All authors read and approved the final manuscript.

## Supplementary Material

Additional file 1 List of EMA approved OMPs (disease) covered in comparative pricing analysis. Click here for file

Additional file 2 Return on Investment simulation. Click here for file
