# Supplementary material for: Sustainable rare diseases business and drug access: no time for misconceptions
Source: Orphanet J Rare Dis. 2013 Jul 23;8:109. doi: 10.1186/1750-1172-8-109 (PMC3735431; doi:10.1186/1750-1172-8-109)
Supplement: Additional file 1 — List of EMA approved OMPs (disease) covered in comparative pricing analysis. [file 1750-1172-8-109-S1.docx]

***Appendix 1 – list of EMA approved OMPs (disease) covered in comparative pricing analysis***

| **Target EU 5 countries are: France, Germany,Italy, Spain, UK** | | | | | |
| --- | --- | --- | --- | --- | --- |
|  |  |  |  |  |  |
| **Rare Diseases drug category** | | **definition** | | | |
| **1st to market non oncology drugs** | | non oncology drugs with no approved medical treatment at time of approval | | | |
| ***Generic name*** | ***Disease*** | ***Generic name*** | ***Disease*** | ***Generic name*** | ***Disease*** |
| Laronidase | MPS I | Mecasermin | Primary insulin-like growth factor-1 deficiency due to molecular or genetic defects | Dornase alfa | Cystic Fibrosis |
| PEG ADA | ADA SCID | Canakinumab | Cryopyrin-Associated Periodic Syndromes (CAPS) | Agalsidase alpha | Fabry disease |
| Carglumic acid | NAGS deficiency | sapropterin dihydrochloride | Hyperphenyl alaninaemia | Eculizumab | PNH |
| imiglucerase | Gaucher disease TI | alglucosidase alfa. | Pompe (late onset) | Bosentan | Pulmonary arterial hypertension (PAH) |
| [Idursulfase](http://www.ema.europa.eu/ema/index.jsp?curl=pages/medicines/human/medicines/000700/human_med_000757.jsp&murl=menus/medicines/medicines.jsp&mid=WC0b01ac058001d124) | MPS II | alglucosidase alfa. | Pompe (infantile) | Tafamidis | transthyretin amyloidosis in adult patients with stage 1 |
| Pirfenidone | Idiopathic pulmonary fibrosis | galsulfase | MPS VI | sodium oxybate. | Cataplexy in adult patients with narcolepsy |
| Deferasirox | Chronic iron overload requiring chelation therapy | romiplostim | Idiopathic thrombocytopenic purpura | miglustat. | Gaucher disease |
| Agalsidase beta | Fabry disease | nitisinone | Tyrosinaemia type 1 | miglustat. | Niemann-Pick disease type C |

| **Target EU 5 countries are: France, Germany,Italy, Spain, UK** | | |  | **Target EU 5 countries are: France, Germany,Italy, Spain, UK** | | |  |  |  |
| --- | --- | --- | --- | --- | --- | --- | --- | --- | --- |
|  |  |  |  |  |  |  |  |  |  |
| **Rare Diseases drug category** | **definition** | |  | **Rare Diseases drug category** | | **definition** |  |  |  |
| **2nd to market non oncology drugs** | non oncology drugs with approved medical treatment in the target indication at time of approval | |  | **Repurposed non oncology rare diseases drugs** | | non oncology rare diseases drugs repurposed in a rare disease indication from a common disease |  |  |  |
| ***Generic name*** | ***Disease*** | |  | ***Generic name (Disease)*** | | ***Disease*** |  |  |  |
| icatibant. | Angioedema | |  | aztreonam | | cystic fibrosis |  |  |  |
| plerixafor | Treatment to mobilize progenitor cells prior to stem cell transplantation | |  | betaine anhydrous. | | Homocystinuria |  |  |  |
| ziconotide | Chronic pain requiring intraspinal analgesia | |  | amifampridine | | Lambert-Eaton myasthenic syndrome |  |  |  |
| eltrombopag | Idiopathic thrombocytopenic purpura | |  | Ibuprofen | | Patent ductus arteriosus |  |  |  |
| pegvisomant | Aacromegaly | |  | Sildefanil | | PAH |  |  |  |
| sitaxentan | PAH | |  | hydroxycarbamide | | Treatment of sickle cell syndrome |  |  |  |
| iloprost | PAH | |  | tobramycin | | Cystic Fibrosis |  |  |  |
| Ambrisentan | PAH | |  | Zinc | | Wilson disease |  |  |  |
| stiripentol. | Severe myoclonic epilepsy in infancy | |  |  | |  |  |  |  |
| rufinamide | Lennox-Gastaut syndrome | |  |  | |  |  |  |  |
| Mannitol | cystic fibrosis (CF) in adults aged 18 years and above | |  |  | |  |  |  |  |
| **Orphan oncology drugs approved by EMA:Target EU 5 countries are: France, Germany,Italy, Spain, UK** | | | | | | | | | |
| ***Generic name*** | ***Disease*** | ***Generic name*** | | | ***Disease*** | | | ***Generic name*** | ***Disease*** |
| Everolimus | advanced renal cell carcinoma | Litak | | | hairy cell leukaemia. | | | Tasigna | chronic myelogenous leukaemia (CML) |
| Arzerra | chronic lymphocytic leukaemia (CLL) | Lysodren | | | advanced (unresectable, metastatic or relapsed) adrenal cortical carcinoma. | | | Tepadina | Conditioning treatment prior to haematopoietic progenitor cell transplantation |
| Atriance | acute lymphoblastic leukaemia | Mepact | | | Osteosarcoma | | | Thalidomide | untreated multiple myeloma |
| Busilvex | Conditioning treatment prior to haematopoietic progenitor cell transplantation | Nexavar | | | hepatocellular carcinoma | | | Torisel | advanced renal cell carcinoma (RCC) |
| [Ceplene](http://www.ema.europa.eu/ema/index.jsp?curl=pages/medicines/human/medicines/000700/human_med_000757.jsp&murl=menus/medicines/medicines.jsp&mid=WC0b01ac058001d124) | acute myeloid leukaemia | Nexavar | | | advanced renal cell carcinoma | | | Torisel | relapsed and/or refractory mantle cell lymphoma [MCL] |
| Evoltra | acute lymphoblastic leukaemia (ALL) | Revlimid | | | multiple myeloma | | | Trisenox | relapsed/refractory acute promyelocytic leukaemia (APL) |
| Gleevec | chronic myeloid leukaemia | Savene | | | anthracycline extravasations | | | Vidaza | acute myeloid leukaemia (AML) |
| Gleevec | malignant gastrointestinal stromal tumours | Sprycel | | | Ph+ acute lymphoblastic leukaemia (ALL) and lymphoid blast CML | | | Vidaza | intermediate-2 and high-risk myelodysplastic syndromes (MDS) |
| Gleevec | chronic eosinophilic leukaemia | Sprycel | | | Philadelphia chromosome positive (Ph+) chronic myelogenous leukaemia (CML) in the chronic phase. | | | Yondelis | advanced soft tissue sarcoma |
| Gleevec | dermatofibrosarcoma protuberans | Sutent | | | Gastrointestinal stromal tumour (GIST) | | | Yondelis | relapsed platinum-sensitive ovarian cancer. |
| Gleevec | acute lymphoblastic leukaemia | Sutent | | | Metastatic renal cell carcinoma (MRCC) | | | Votubia | subependymal giant cell astrocytoma (SEGA) associated with tuberous sclerosis complex (TSC) |
| Gleevec | myelodysplastic/myeloproliferative diseases (MDS/MPD) | Sutent | | | Pancreatic neuroendocrine tumours (pNET) | | |  |  |
