# Supplementary material for: Sustainable rare diseases business and drug access: no time for misconceptions
Source: Orphanet J Rare Dis. 2013 Jul 23;8:109. doi: 10.1186/1750-1172-8-109 (PMC3735431; doi:10.1186/1750-1172-8-109)
Supplement: Additional file 2 — Return on Investment simulation. [file 1750-1172-8-109-S2.pdf]

Appendix 2 –Return on Investment simulation

**Baseline benchmark**	Base case e-IRR	
**Estimated late stage portoflio ROI (cost of capital)**	**11.0%**	**14.0%**
R&D spend (M $)	$65	$65
Post tax earnings 10 y later	$185	$241
tax rate	35%	35%
10 y Pre tax earnings (M $)	$284	$371
trading margin (excl R&D)	40%	40%
required incremental sales 10 years later(M $)	**$473**	**$618**

**Rare diseases Simulation Case study**		
Estimated late stage portoflio ROI (cost of capital)	**11.0%**	**14.0%**
Rare diseases companies annual R&D cumulated spend 2011 (M $)(*)	**$2 871**	**$2 871**
Post tax earnings 10 y later	$8 153	$10 645
tax rate	35%	35%
10 y Pre tax earnings (M $)	$12 543	$16 376
trading margin (excl R&D)	40%	40%
required incremental sales 10 Y later	**$20 905**	**$27 294**
(*) Actelion, Alexion, Biomarin, Dyax, Enzon,Genzyme,Shire, Sobi, United Therapeutics, Viropharma		
Corresponding 10 y CAGR	***5%***	***8%***
